# Supplementary figures and images for: Wildlife vaccination strategies for eliminating bovine tuberculosis in white-tailed deer populations
Source: PLoS Comput Biol. 2024 Jan 4;20(1):e1011287. doi: 10.1371/journal.pcbi.1011287 (PMC10793927; doi:10.1371/journal.pcbi.1011287)

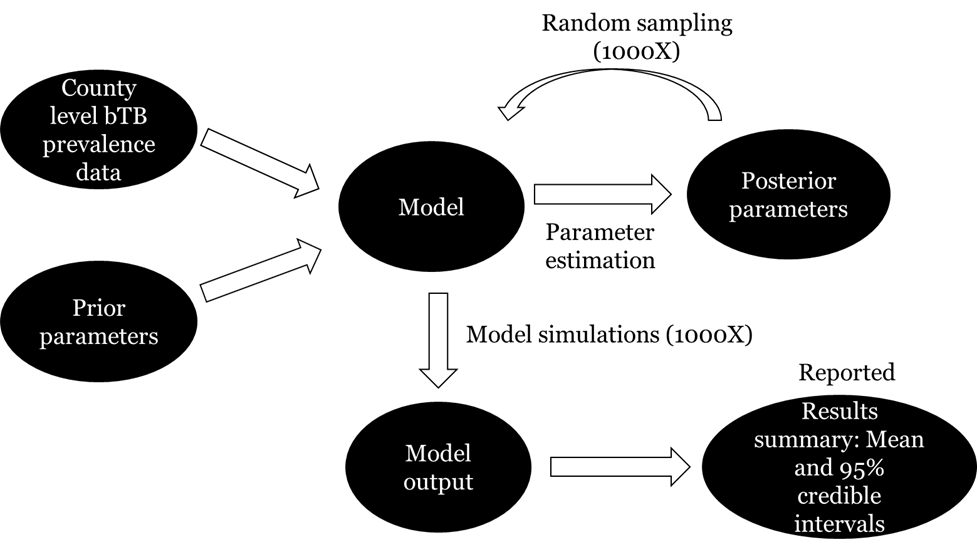

Supplement: S1 Fig — (TIF) [file pcbi.1011287.s001.tif]

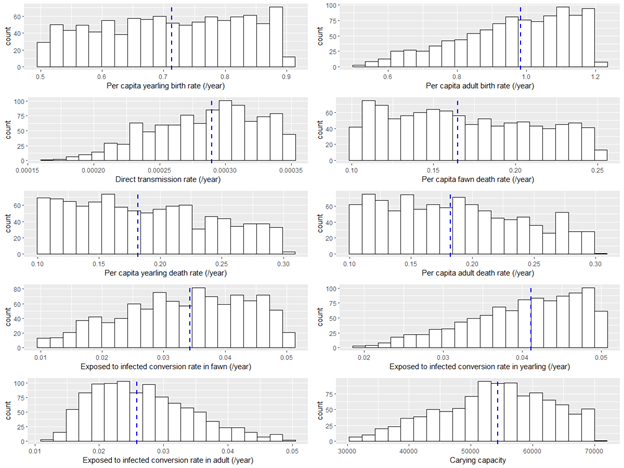

Supplement: S2 Fig — (TIF) [file pcbi.1011287.s002.tif]

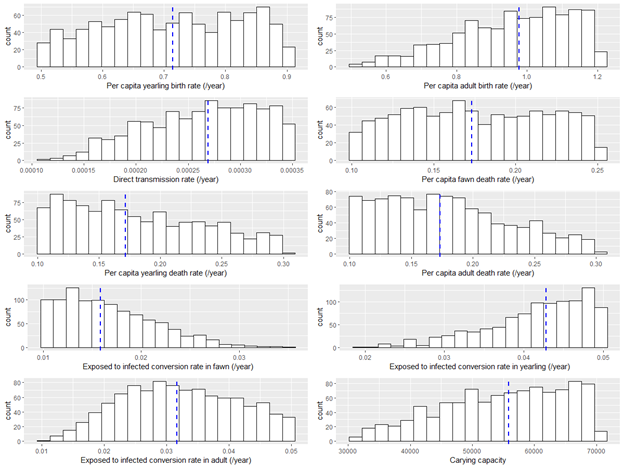

Supplement: S3 Fig — Distribution shows 1000 estimated parameter values using approximate Bayesian computation. Dashed vertical lines show median values from the distribution. (TIF) [file pcbi.1011287.s003.tif]

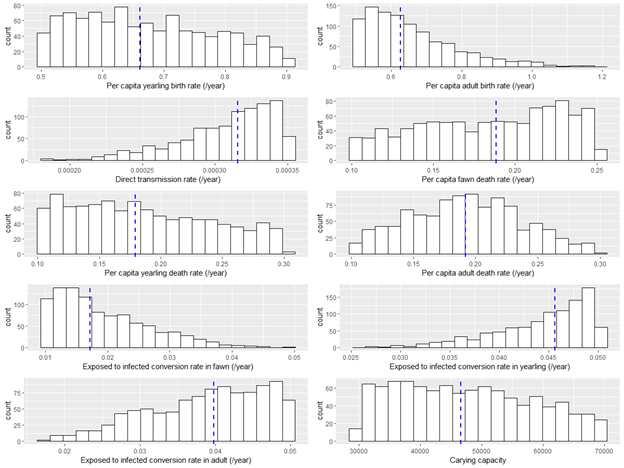

Supplement: S4 Fig — Distribution shows 1000 estimated parameter values using approximate Bayesian computation. Dashed vertical lines show median values from the distribution. (TIF) [file pcbi.1011287.s004.tif]

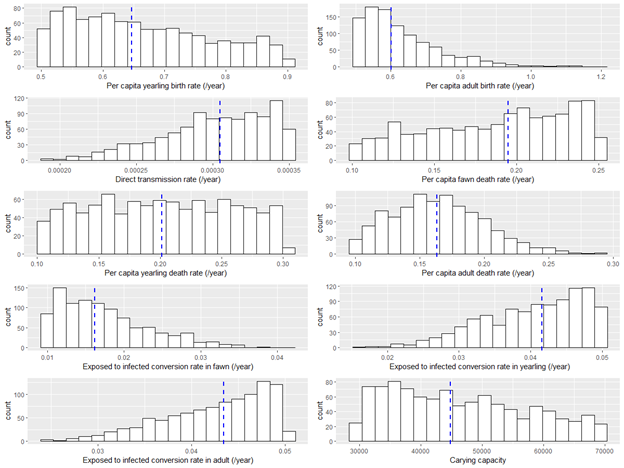

Supplement: S5 Fig — Distribution shows 1000 estimated parameter values using approximate Bayesian computation. Dashed vertical lines show median values from the distribution. (TIF) [file pcbi.1011287.s005.tif]

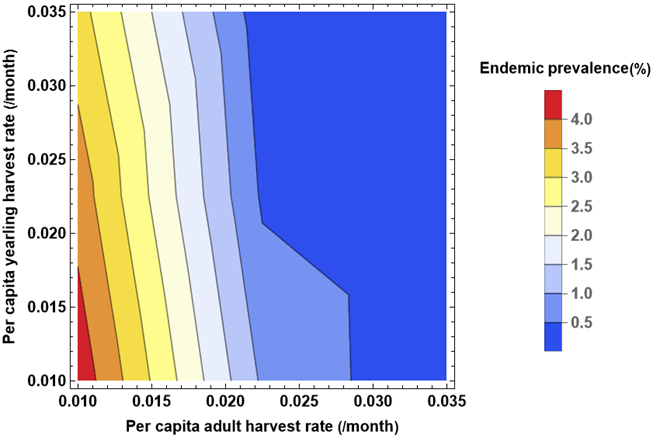

Supplement: S6 Fig — All other parameters are median estimated parameters for Alpena County Michigan assuming no vaccination. See Table 1 for the parameters used. (TIF) [file pcbi.1011287.s006.tif]

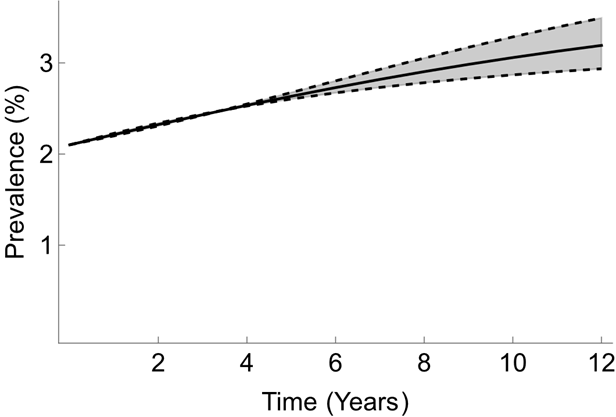

Supplement: S7 Fig — Upper dashed line shows scenario when adult deer harvest rate is decreased by 50% and lower dashed line shows scenario when the adult harvest rate is increased by 50%. Solid line represents baseline projection with no change in harvest rate. All other parameters used for the Figs are estimated median parameter values for Alpena County. (TIF) [file pcbi.1011287.s007.tif]

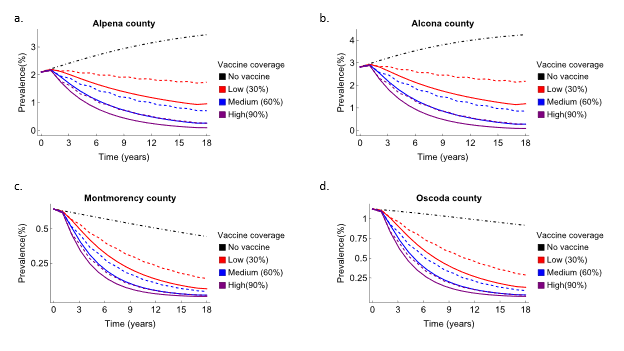

Supplement: S8 Fig — Vaccination was applied from year 1 to year 16. Black dashed and dotted line represent baseline scenarios with no vaccination. Continuous lines show prevalence for yearly vaccination at different vaccine coverage. Dashed lines show prevalence assuming biennial vaccination for the same time. All simulations for each county were done using median estimated parameter values. (TIF) [file pcbi.1011287.s008.tif]

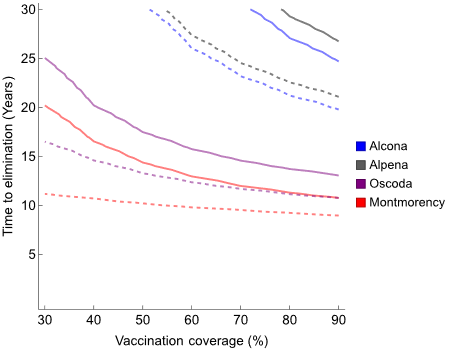

Supplement: S9 Fig — Gray, blue, purple, and red represent Alpena, Alcona, Oscoda, and Montmorency counties in Michigan, respectively. Continuous lines represent time of elimination with current level of adult harvest whereas dashed lines represent the time of elimination when harvest is increased by 30% of the current values. Other vaccine parameters include ϵL = 0.2, κ = 0.5, and α = 0.06. (TIF) [file pcbi.1011287.s009.tif]

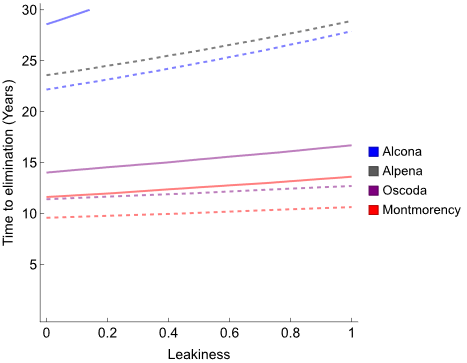

Supplement: S10 Fig — Gray, blue, purple, and red represent Alpena, Alcona, Oscoda, and Montmorency counties, respectively. Continuous lines represent time of elimination with current level of adult harvest whereas dashed lines represent the time of elimination when harvest is increased by 30% of the current values. Other vaccine parameters include p = 0.7, κ = 0.5, and α = 0.06. (TIF) [file pcbi.1011287.s010.tif]

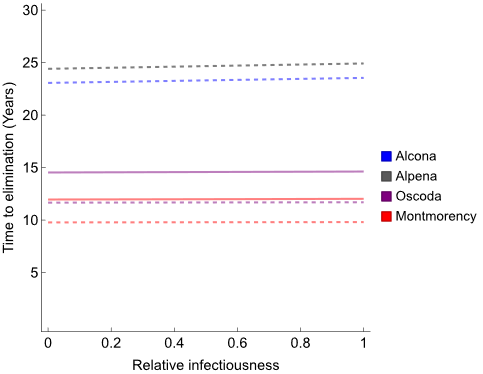

Supplement: S11 Fig — Black, blue, purple, and red represent Alpena, Alcona, Oscoda, and Montmorency counties, respectively. Continuous lines represent time of elimination with current level of adult harvest whereas dashed lines represent the time of elimination when harvest is increased by 30% of the current values. Other vaccine parameters include ϵL = 0.2, p = 0.7, and α = 0.06. (TIF) [file pcbi.1011287.s011.tif]

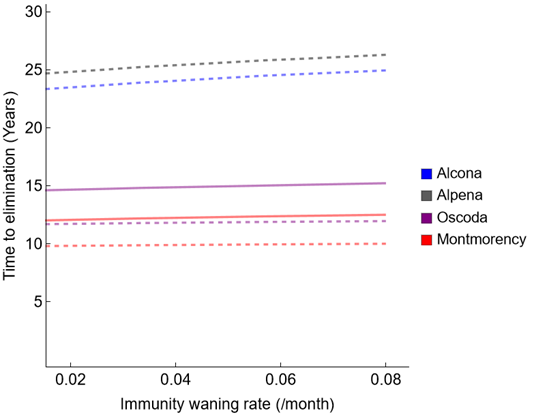

Supplement: S12 Fig — Black, blue, purple, and red represent Alpena, Alcona, Oscoda, and Montmorency counties, respectively. Continuous lines represent time of elimination with current level of adult harvest whereas dashed lines represent the time of elimination when harvest is increased by 30% of the current values. Other vaccine parameters include ϵL = 0.2, p = 0.7, and κ = 0.5. (TIF) [file pcbi.1011287.s012.tif]

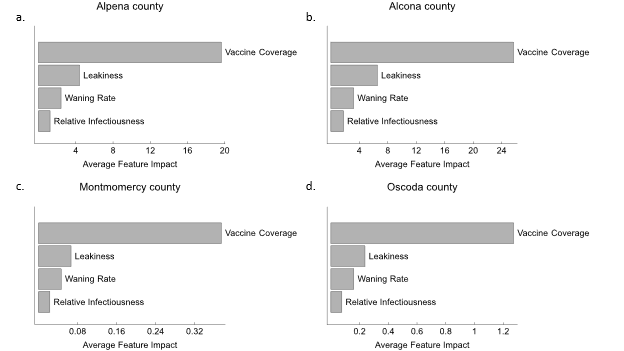

Supplement: S13 Fig — Estimated median parameter values for each county is used. See method section for the range of vaccine parameters used to obtain the results. (TIF) [file pcbi.1011287.s013.tif]

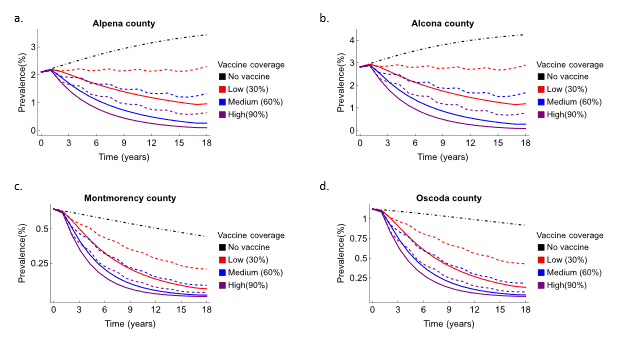

Supplement: S14 Fig — Vaccination was applied from year 1 to year 16. Black dashed and dotted line represent baseline scenarios with no vaccination. Continuous lines show prevalence for yearly vaccination at different vaccine coverage. Dashed lines show prevalence assuming vaccination every 3 years for the same time frame. All simulations for each county are done using median estimated parameter values. (TIF) [file pcbi.1011287.s014.tif]

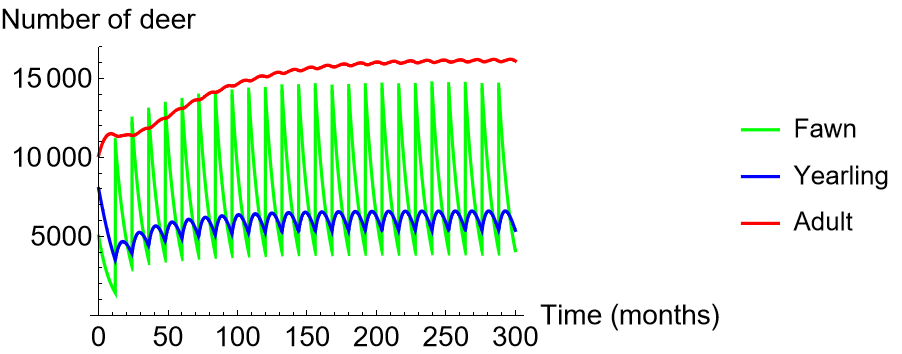

Supplement: S15 Fig — The red line shows the number of adult deer, blue line represents number of yearling deer and green shows number of fawns. The fluctuation is due to birth pulses. The deer number estimates are obtained from the main model using median estimated parameter values. (TIF) [file pcbi.1011287.s015.tif]

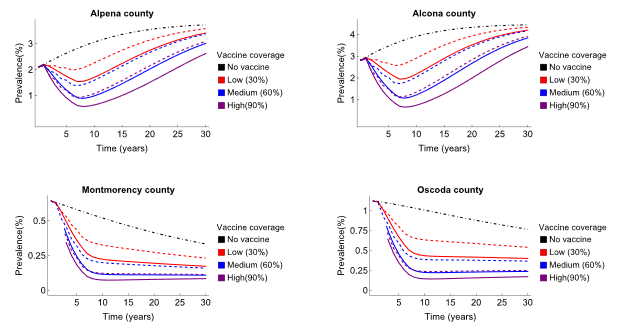

Supplement: S16 Fig — Vaccination was applied from year 1 to year 6. Black dashed and dotted lines represent baseline scenarios with no vaccination. Continuous lines show prevalence for yearly vaccination at different vaccination coverage. Dashed lines show prevalence assuming biennial vaccination for the same time. All simulations for each county were done using median parameter estimates. Other vaccine parameter values included ϵL = 0.2, κ = 0.5, and α = 0.06. (TIF) [file pcbi.1011287.s016.tif]

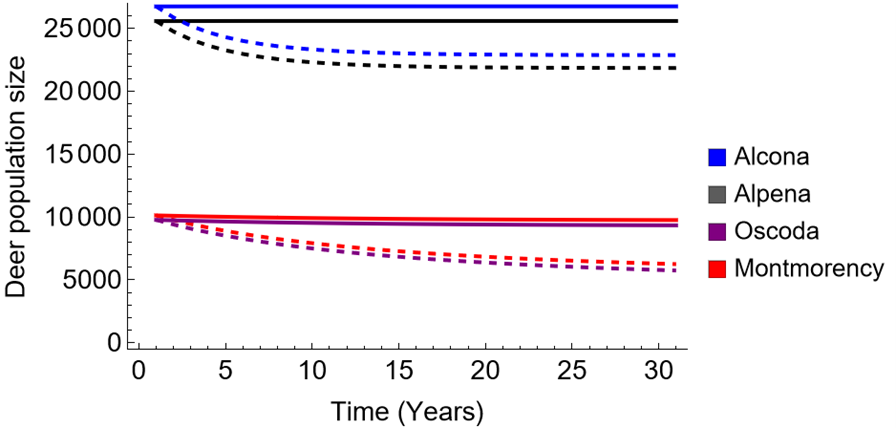

Supplement: S17 Fig — Continuous lines represent deer population with current level of harvest rates whereas dashed lines represent deer population when the harvest rate is increased by 30%. We assumed vaccine coverage to be 70% and yearly vaccination for 30 years. All other parameters are the same as in Fig 5A. (TIF) [file pcbi.1011287.s017.tif]

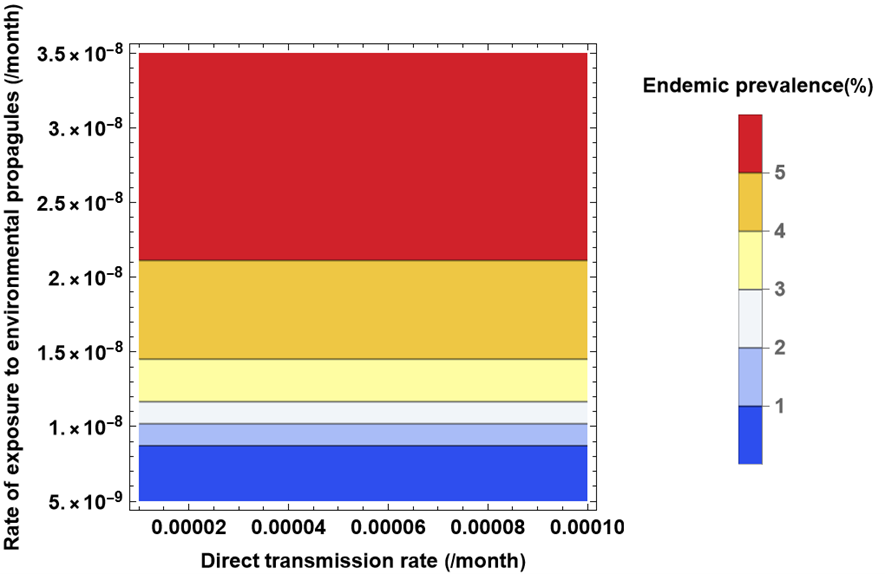

Supplement: S18 Fig — All other parameters are estimated median parameters for Alpena County. (TIF) [file pcbi.1011287.s018.tif]

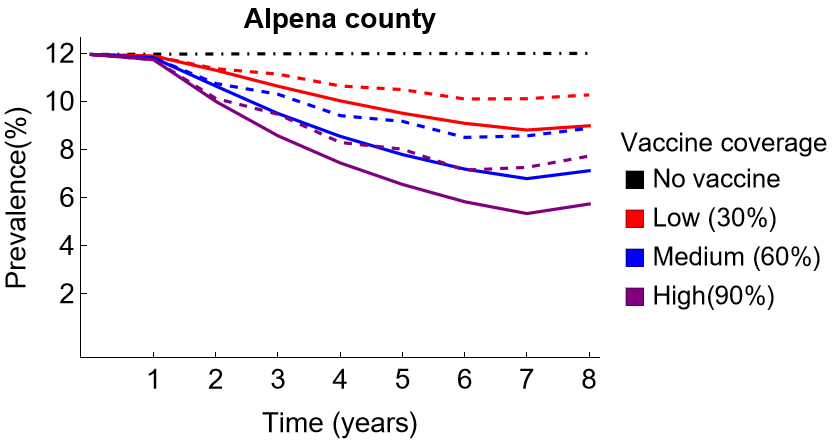

Supplement: S19 Fig — The parameters were estimated assuming 5X that the ’true prevalence’ is 5X the observed apparent prevalence. Vaccination was applied from year 1 to year 6. Black dashed and dotted lines represent baseline scenarios with no vaccination. Continuous lines show prevalence for yearly vaccination at different vaccination coverage. Dashed lines show prevalence assuming biennial vaccination for the same time. All simulations for each county were done using median parameter estimates. Other vaccine parameter values included ϵL = 0.2, κ = 0.5, and α = 0.06. (TIF) [file pcbi.1011287.s019.tif]

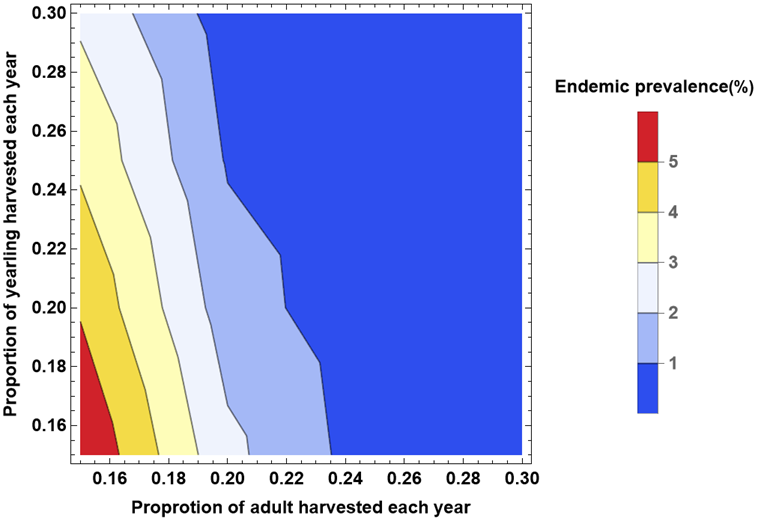

Supplement: S20 Fig — For this, we assume that a proportion of a given age class of deer are harvested during a month. More specifically, we assume it occurs during December every year. Transmission rates are adjusted to fit the data to the modified model. All other parameters are median estimated parameters for Alpena County Michigan assuming no vaccination. See Table 1 for the parameters used. (TIF) [file pcbi.1011287.s020.tif]

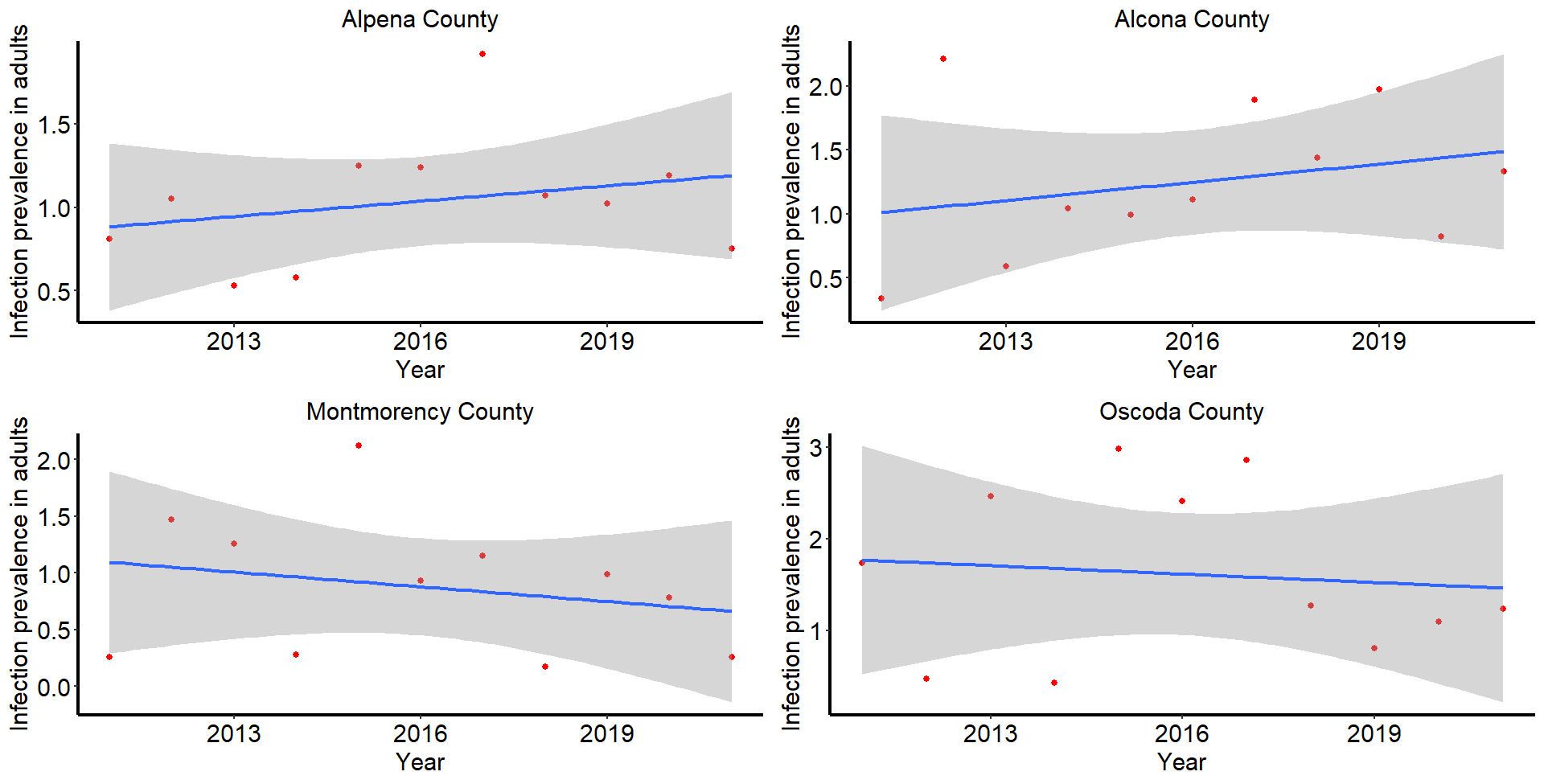

Supplement: S21 Fig — (TIF) [file pcbi.1011287.s021.tif]
