# Supplementary material for: Wildlife vaccination strategies for eliminating bovine tuberculosis in white-tailed deer populations
Source: PLoS Comput Biol. 2024 Jan 4;20(1):e1011287. doi: 10.1371/journal.pcbi.1011287 (PMC10793927; doi:10.1371/journal.pcbi.1011287)
Supplement: Attachment — Submitted filename: Response to reviewers.docx [file pcbi.1011287.s026.docx]

**Response to reviewer’s comments:**

Reviewer's Responses to Questions

**Comments to the Authors:
 Please note here if the review is uploaded as an attachment.**

Reviewer #1: Pandey et al. have used a modeling approach to assess vaccination strategies for eliminating bTB in Michigan white-tailed deer populations. This topic is very relevant to managing a serious wildlife health, public health and economic issue, and timely. I have the following suggestions/comments:

Thank you for your kind assessment. Please see below for detailed responses to each question and comment - we hope these revisions meet your approval.

1) bTB surely is an excellent example of a pathogen transmitted at the wildlife-domestic-human interface, but your work exclusively focuses on the wildlife component - you have evaluated strategies for bTB elimination /control in WTD populations, right? I suggest replacing the 'wildlife-livestock interface' in your title to 'white-tailed deer populations'.

Thank you for your suggestion. We have now modified the title to include white-tailed deer populations.

2) You compare model results for 4 counties - yet there is no countywise deer population data provided in your paper. How does the N differ between these counties? How does the age composition differ between these counties? Did you use pre-harvest or post-harvest estimates?

Thank you for these questions. It is correct that we have not explicitly shown deer population size in these counties. However, we estimated the theoretical carrying capacity of each county (Figure S2-S4). This and other parameters determine the deer population dynamics, which fluctuates because of birth pulses and continuous death and harvest. As a result, the age composition also fluctuates throughout the year. An example of how deer population dynamics fluctuates in Alpena County is now added in supplementary figure (Figure S15) to address this comment.

In the model, we have assumed constant/continuous harvest of deer throughout the year for simplification. This is further supported by the fact that the USDA removes deer outside of the hunting season. Hence, there is no direct estimate of pre- / post-harvest deer numbers. But since the vaccination is done just before the birth pulses, we can say that vaccination applies to ‘post-harvest’ deer numbers. But this can lead to confusion as the harvest is assumed to be continuous. Thus, we avoided pre- / post-harvest terms.  That said, in the revision we now explicitly acknowledge our model assumption in lines 177-180.

3) You have provided parameter values in Table 1. All except the per capita immune waning rate are yearly (annual) rates. How do you account for this in your model?

Most rates are assumed to be constant throughout the year. The annual rates can be converted to monthly rates by simply dividing them by 12 (except for birth rates, since these are pulses). To avoid confusion, the monthly rate is now shown as the yearly rate (Table 1).

4) Moreover, you have assumed that per capita harvest rates are same for male and female deer. Could you provide some explanation why you made this assumption when the harvest rates in the real world are clearly different in male and female deer? Do consider that bTB prevalence differs significantly between males (8%) and females (2%) as documented by O'Brien et al 2002. I might have missed any current references on this topic; if so, please include these references.

Thank you for this comment. We agree that this is a valid concern. Theoretically, we could have further expanded the model to include sex differences in harvest rates and disease prevalence. But since we are already dealing with a big deterministic model with limited knowledge of parameters, the model is unidentifiable. Hence, we had to fix some of the parameters to estimate the remaining parameters. We already have a low resolution on parameter estimates. If we include sex differences, the number of equations roughly doubles and so does the number of parameters needed to be estimated. This further complicates parameter estimation and model prediction, making it difficult to have any sensible predictability. Thus, we had to make the simplifying assumption of equal rates in male and female deer to estimate the parameters. This allows us to make predictions guided by the available data. We now acknowledge the model limitation in lines 453-455, along with the reference to O’Brien et al., 2002 in the following sentence. Additional data and knowledge on disease and population dynamics would allow for a more detailed model. However, this is an area of further research in the future to enhance predictability.

5) This also brings up the question about the sensitivity analysis - you state that disease prevalence is very sensitive to adult harvest rates. But you make unrealistic assumption about male and female harvest rates. How valid are your conclusions in the context of this assumption?

As we mentioned in the previous response, simplifications were necessary to make the parameter estimation possible. This includes the assumption of equal harvest rates of male and female deer. Greater female harvest rates would decrease the deer number in the current generation and have a more drastic effect in following generations. As a result, this can result in a greater reduction in disease prevalence compared to what we have shown. Hence, our model underestimates potential reduction in prevalence. We now acknowledge and describe this in our model limitation section (lines 462-465).

 6) Line 290: Should'nt it be Figure 2?
 Thank you for catching the typo. It is now corrected.

7) Another critical component missing from your results is the impact of these scenarios on the deer population. For scenarios where harvest and vaccination are combined, could you provide a plot to show the population size of deer? Would help the reader understand the context for these results.

Excellent idea, a figure showing the deer population size when harvest and vaccination are combined is now added in the supplemental file (Figure S17) and a reference is made in the results section (Line 366-367). Vaccination does not affect deer population size, as we assumed no death from the disease. Increased harvest leads to reduction in estimated deer population sizes as expected.

 8) Figure 4 would provide critical insights if you extend the x axis (years) to 30? How long does the effect of short term vaccination last?

Agreed, we have now added a figure in the supplemental file (Figure S16). We show that short term vaccination efforts (of interest to the funding agency) can have a long-lasting effect on disease prevalence trends and make a reference to this in the results section (Lines 342-343).

Reviewer #2: The authors develop an age structured mechanistic model that is fitted on bovine TB surveillance data in white tailed deer in Michigan. They use the models to test effect vaccines (different efficacy and coverage) and deer harvesting on bTB transmission dynamics. The manuscript is beautifully written with a good flow of logic in the introduction, and good explanation of the methods as well as good presentation of the results, and insights drawn from the model. I enjoyed reading it.

Thank you for your kind remarks-they are much appreciated.

I have two main comments for consideration by the authors:
 - What is relative contribution of live propagules in the environment to the main outcome modelled (prevalence of bTB)? This number of live propagules in the environment would be expected to be a function of prevalence of bTB in the study population - how is this accounted for in the model?

Thank you for these questions. Given the current state of knowledge and the available data on bTB dynamics, it is difficult to make confident estimates of the relative contribution of direct vs indirect transmission to disease prevalence. More data on environmental shedding rate and persistence rate of environmental propagules are needed to make confident inference. For modeling purposes, we assumed, based on previous literature, that most of the transmission in white-tailed deer is due to direct transmission. Sensitivity analysis for the current model shows that it is more sensitive to environmental transmission rate compared to direct transmission. We have now added this to the supplemental figure (Figure S18).

As for the second question, the number of live propagules is a function of the number of infected individuals (and hence bTB prevalence). This is already accounted for in the model (line 257) where the rate of change of propagule number in the environment is dependent upon the shedding from infected individuals and the decay rate of propagules.

- The authors rightfully identify sampling bias as a potential limitation to the data used to parametarize the model. I would be keen to see how the model results and interpretation of insights would vary if under ascertainment of the infection rates was incorporated in the model.

Thank you for these suggestions. We considered scenarios where the estimated prevalence was higher than the observed/apparent prevalence in Alpena County. But we did not report it for several reasons. First, we did not have data to estimate how different the actual prevalence would be from the apparent prevalence. We were able to estimate detection probabilities at the quarter section level (work under review in another outlet), which highlighted that apparent prevalence is likely lower than true prevalence, but those estimates of detection probability do not inform true prevalence directly. Thus, we opted to fit the model to apparent prevalence estimates for this work and explore how robust the results would be to different magnitudes of prevalence. When we estimated parameter values assuming 5-fold differences in apparent prevalence for Alpena County, most trends observed with current apparent prevalence shown in the manuscript held even with a different prevalence assumption. But, the magnitudes were larger with increased prevalence. We opted not to include this result in the manuscript, as the prevalence estimates we used to explore model robustness were completely hypothetical. However, we now added a sentence describing that we tested the model under different prevalence levels and that the relative results were unchanged (Lines 462-465)-. We also added one hypothetical scenario in the supplemental figure to show the effect of short-term vaccination in the disease prevalence. (Figure S19).
