# Supplementary material for: Wildlife vaccination strategies for eliminating bovine tuberculosis in white-tailed deer populations
Source: PLoS Comput Biol. 2024 Jan 4;20(1):e1011287. doi: 10.1371/journal.pcbi.1011287 (PMC10793927; doi:10.1371/journal.pcbi.1011287)
Supplement: Attachment — Submitted filename: Response to reviewers.docx [file pcbi.1011287.s027.docx]

**Response to reviewer:**

Dear Dr. Pandey,

Thank you very much for submitting your manuscript "Wildlife vaccination strategies for eliminating bovine tuberculosis in white-tailed deer populations" for consideration at PLOS Computational Biology.

As with all papers reviewed by the journal, your manuscript was reviewed by members of the editorial board and by an independent reviewer. In light of the review (below this email), we would like to invite the resubmission of a significantly-revised version that takes into account the reviewer's comments.

Please carefully address the critical comment 1 of the reviewer, which might lead to methodological problems. It may also be helpful to implement a sensitivity analysis testing for the robustness of such an assumption/procedure.

Thank you for your comments and suggestions. We have now revised the manuscript based on suggestions. Specifically, we added two figures (figs S20, S21) to the supplement showing the sensitivity analysis of our assumption. As discussed below in detail, there are no significant differences in the predicted patterns of disease dynamics even with modified assumptions (fig S6 vs fig S20, Line 329), providing support that our method is justified. Please see below for specific changes and responses to the reviewer’s comments.

Reviewer #1: Thank you for responding to my comments. I have a few follow-up questions:

Thank you for your comments and suggestions. We have modified the manuscript and added two figures to the supplement materials. Please see below for a more specific response.

1) You state in your response to Comment 2: "In the model, we have assumed constant/continuous harvest of deer throughout the year for simplification. This is further supported by the fact that the USDA removes deer outside of the hunting season." I think this simplistic assumption is problematic. Just considering the harvest data for Alpena County, about 10% of the deer (~2500) are removed (harvested) in the month of November; a total of ~15% (3500) removed during the 4 months of harvest season (Sep-Dec). But, as per your explanation, you are simply dividing the annual harvest rate by 12 to get a monthly harvest rate, so you have 400 deer harvested every month in your model. The sensitivity analysis (Fig. S6) is questionable due to the unrealistic monthly harvest rates. Such simplifying assumptions influence model dynamics and disease dynamics, and in my opinion, negatively impact the applicability and utility of your modeling approach.

Thank you for your comments. In the model, we assumed age-specific continuous and instantaneous harvest rates, so they do not directly correspond to the % or numbers provided above. But the overall total deer harvested within a year is close to the actual numbers of deer harvested in Alpena County. As mentioned in the manuscript, we made the simplifying assumption of continuous harvest with instantaneous per capita rates. While this is different from the actual practice where deer are harvested at differential rates each month, having simplified harvest rates makes it easier for us to fit the model to disease prevalence data and to explore other features of the model. Additionally, since bovine TB is a slow progressing and chronic disease, we believe the heterogeneous processes that occur at faster time scales can be smoothed out while looking at disease dynamics that occur at longer timescales. Nevertheless, we tested whether having a different harvest structure would have an effect in disease prevalence patterns. For that, we assumed that a fraction of deer from each class are harvested during a specific month (December) with no harvest during other months (Lines 180-182 in revised text). Even with this different harvest structure, we see similar sensitivity patterns (Fig S20) and our assertion using the simplified model that disease prevalence is more sensitive to adult harvest rates is still valid (Line 329).

2) Do consider converting the finite rates to instantaneous rates instead of just dividing annual rates by 12. See<https://influentialpoints.com/Training/finite-and-instantaneous_rates.htm>

Thank you for this suggestion. The rates we considered in the model are indeed instantaneous rates, except for vaccination and birth rates that are assumed as pulses and hence are similar to finite rates. Other rates such as harvest (in the main model), death, disease progression etc., are instantaneous.

3) You state that (Line 305) "Prevalence increased in Alpena and Alcona counties over the ten-year period, remained level in Oscoda County and decreased in Montmorency County." Is this based on 'eyeballing' Figure 2, or you used some other statistic? Please provide details here.

This was based on the median prevalence values from the model. We now have added a linear fit to the apparent prevalence data as well (Fig S21). Both show similar trends (Lines 310-311).

4) Similarly, you state (Line 324) "Prevalence was most affected by increase (30%) harvest in Alpena and Alcona counties, whereas very little effect was seen in Montmorency and Oscoda counties." How was this ('most affected', 'little effect') determined? Did you do statistical comparisons? If so, please provide details.

Thank you for the suggestion. To back up our assertion, we have now included the data on reduction (Lines 332- Lines 335). These are estimates based on model prediction at the end of the simulation year (Year 12).

5) It is surprising that you did not discuss in more detail the decreasing prevalence of bTB in Oscoda and Montmorency counties (Figures 3, 4, S16, etc.). As you are using a compartmental model, this means the R0 is less than 1, but in the context of Fig. S17, it might just be due the population threshold size (Nt). This might not reflect real-world process, and majorly affects the relevance of your findings.

Thank you for these suggestions. We have now added more details about decreasing prevalence of bTB in Oscoda and Montmorency (Lines 316-320, and Lines 413-415). Although we briefly discussed a possible reason for why bTB prevalence is decreasing, we cannot confidently attribute this to a specific parameter/process because of low parameter resolution in posterior distributions (Lines 314-316). Further research on this disease system will probably be able to identify factors that are driving differential disease dynamics in these four counties.
